# Editorial: The biological impact of adversity on cardiovascular disease risk and obesity

**DOI:** 10.3389/fcvm.2022.1022724

**Published:** 2022-09-21

**Authors:** Yvonne Baumer, Nicole Farmer, Alika K. Maunakea, Belinda L. Needham, Tiffany M. Powell-Wiley

**Affiliations:** ^1^Social Determinants of Obesity and Cardiovascular Risk Laboratory, Cardiovascular Branch, Division of Intramural Research, National Heart Lung and Blood Institute, National Institutes of Health, Bethesda, MD, United States; ^2^Translational Biobehavioral and Health Disparities Branch, National Institutes of Health, Clinical Center, Bethesda, MD, United States; ^3^Department of Anatomy, Biochemistry and Physiology, John A. Burns School of Medicine, University of Hawaii, Honolulu, HI, United States; ^4^Department of Epidemiology, University of Michigan, Ann Arbor, MI, United States; ^5^Intramural Research Program, National Institute on Minority Health and Health Disparities, National Institutes of Health, Bethesda, MD, United States

**Keywords:** social determinants of health, cardiovascular disease, obesity, adversity, cardiovascular risk factors

The COVID-19 pandemic and the spotlight on racial injustices over the last 2 years have brought renewed attention to the social determinants of health (SDoH), defined as the economic, social, environmental, and psychosocial factors that influence health (1). Cumulative and repeated exposures to adverse SDoH, including discrimination/racism, under-resourced neighborhoods, unsafe housing, and limited access to adequate education and income, serve as sources of chronic psychosocial stress and are linked to the development of cardiometabolic diseases including obesity, diabetes, and cardiovascular disease (CVD). Chronic adversity related to SDoH can activate pathogenic molecular mechanisms and pathways, including hyperstimulation of sympathetic nervous activity (SNA) or the hypothalamic-pituitary-adrenal axis (HPA), which promote the development of CVD and related risk factors (1). However, further research is needed to identify specific signaling pathways and cellular functions that connect psychosocial and environmental stressors to cardiometabolic disease, which may elucidate future multi-level intervention targets. This current special edition entitled, “*The Biological Impact of Adversity on Cardiovascular Disease Risk and Obesity*,” was designed to feature innovative studies and reviews that advance knowledge of biological mechanisms by which chronic psychosocial and environmental adversity may promote CVD risk over the life course.

## Role of childhood psychosocial exposures and CVD risk

Four articles in this special edition highlight the role of childhood adversity in the development of CVD risk into adulthood. Salzmann et al. reviewed how childhood maltreatment (CM), specifically emotional neglect or the failure to meet a child's need, can promote psychological distress, depression and anxiety, which subsequently leads to an increased incidence of cardiovascular risk factors and CVD with or without the involvement of CVD-accelerating behavioral changes. For their review, Salzmann et al. provide a heuristic model of how CM relates to CVD risk factors and comorbidities and offer suggestions for future research including distinguishing between emotional neglect and abuse; evaluating and identifying how CM impacts vulnerable patients; and encouraging providers to conduct CM screenings.

Utilizing the Trier social stress test (TSST) as an acute stressor, Tell et al. showed that higher levels of childhood physical abuse reported by African-American men were associated with lower heart rate variability during the TSST. Lower heart rate variability, as measured by a higher LF/HF ratio (low frequency to high frequency heart rate ratio), among participants with greater exposure to emotional and physical abuse was associated with an accelerated pro-inflammatory IL-6 response to the TSST. Similar to the Salzmann et al. review, this study suggests that childhood abuse and neglect associate with pro-inflammatory hyperreactivity in adults potentially through altered autonomic nervous system (ANS) responses to stress. This study and those in the review demonstrate the need to characterize the role of abuse and neglect experiences in studies evaluating stress and inflammation among adults, and the need for screening for childhood abuse and neglect among pediatric as well as adult patient populations.

Additionally, Lei et al. show the biological embeddedness of social processes in CVD risk using longitudinal data from adolescents aged 10–29 years in the Family and Community Health Study (FACHS). This study assessed the role of harsh parenting, defined by self-report of a primary caregiver engaged in shouting, criticizing, lecturing, or physical aggression, in incident weight gain, and evaluated DNA methylation as a mediator between the social process and obesity. Using DNA methylation analysis at five CpG index sites associated with obesity, the authors found that methylation of these sites mediated not only the relationship between harsh parenting and obesity but also the relationship between harsh parenting and expression of obesity-related genes at the corresponding CpG sites. Ultimately, results from this and other studies in this issue focused on childhood abuse and neglect support preventive strategies among children and adolescents to limit the effects of emotional and physical abuse on CVD risk, which can have lasting adverse health impacts.

In contrast to the prior studies, Ding et al. evaluated the role of nutritional famine exposure to CVD risk using data from the prospective, China-based Kailun study of adults. In their study, famine, an extreme scarcity of food often due to war, natural disaster, crop failures, poverty, government policies or economic catastrophes, was measured by a proxy variable of birth year blocks to create a fetal-exposed group, a childhood-exposed group, and non-exposed group. The authors found that exposure for the fetal group was associated with incident CVD over an 11 year follow-up period. Furthermore, the authors found a protective modification effect for the presence of ≥3 adapted ideal cardiovascular health metrics (non-smoking, BMI < 24 kg/m^2^, physical activity at least 80 min per week of moderate-intensity physical activity, daily salt intake < 6 g, total cholesterol < 5.2 mmol/L, BP < 120/80 mmHg, fasting blood glucose (FBG) < 5.6 mmol/L) for males in the fetal-exposed group, but not females. Future research should investigate the potential driving factors and underlying signaling pathways that may explain the observed sex-specific differences.

## Role of the neighborhood environment on the biology of adversity

Additional studies in this issue highlight the role of neighborhood environment on mechanistic pathways associated with CVD development. In the Multi-Ethnic Study of Atherosclerosis (MESA), Wang et al. demonstrate that individual- and neighborhood-level disadvantage are associated with monocyte DNA methylation states and subsequent cardiovascular risk factors like BMI, insulin, or HDL-C Further analyses found that the methylation states at more than 400 CpGs were significant mediators between cardiovascular risk factors and both individual and neighborhood SES; many of genes associated with these CpGs were involved in antigen processing and presentation. However, when controlling for BMI, the majority of candidate CpG sites lost significance, suggesting that BMI is a part of the epigenetic mediation pathway. These findings highlight that epigenetic modification may function as a mediator between socioeconomic disadvantage and CVD risk. Overall, the monocyte-specific results provide further evidence for the role of monocyte activation and function in CVD risk.

Given the increasing evidence demonstrating mechanistic linkages between environment and CVD risk, could behavioral interventions modify this risk? Park et al. utilized data from MESA and the Jackson Heart Study (JHS) to examine the importance of self-reported optimism on cardiovascular health outcomes. Park et al. used composites based on Life's Simple 7, including smoking, BMI ≥30, sedentary behavior, poor diet intake, total cholesterol ≥ 240 mg/dl, elevated blood pressure, and elevated glucose. The authors found that optimism positively associated with better adherence to Life's Simple 7, but the associations were impacted by specific psychosocial and environmental risks, such as chronic stress and neighborhood deprivation. Future studies should examine potential multi-level interventions that reduce neighborhood-level disadvantage and promote optimism while concurrently examining the effects of these interventions on biological pathways like epigenetic modifications and cellular function.

Sunderraj et al. further highlight the role of neighborhood exposures in heart failure as a specific CVD condition. Using the Centers for Disease Control measure of Social Vulnerability Index, which is a composite of 15 social factors, including minority status, disability status, unemployment, and educational attainment, the authors found that individuals without a known clinical diagnosis of heart failure who were from residential areas with a higher SVI score had an increased risk of myocardial fibrosis than individuals from lower SVI areas (Sunderraj et al.). These tissue-level observed changes should be explored in future studies to determine how neighborhood-related factors, such as food and built environment that limit healthy dietary intake and physical activity, respectively, could signal increased extracellular matrix production by cardiac cells and subsequent cardiac fibrosis.

Relatedly, individual level and institutional racism, racial and class segregation, and discrimination in housing promote stress and cardiometabolic risk. A final review in this Research Topic examines how housing-related SDoH are associated with the biology of adversity (Sistrunk et al.). Reviewing three key societal and structural factors: “redlining,” (systematic denial of services of loans, insurance policies, park and grocery store services to residents of communities based on race and/or ethnicity), zoning (differential industrial vs. residential zoning based on race/ethnicity predominance of the zoned area), and the deliberate routing of highway construction within communities of color, the authors link these factors with biological processes such as telomere shortening, allostatic load, oxidative stress, and tissue inflammation. In relation to CVD risk, the authors also explored the impact of inflammation on the immune system and the molecular mechanisms by which an inflamed immune microenvironment is related to the atherosclerotic plaque development. Research studies such as these by Sistrunk et al. and Sunderraj et al. further emphasize the need for macro-level interventions that acknowledge the historical impacts of discriminatory policies in housing and the need to address the neighborhood-related health risks that persist.

## Future perspectives

Interdisciplinary research that combines both population-based studies with translational science can examine the mechanisms by which psychosocial and environmental factors as SDoH lead to CVD development (2). By understanding the adverse biological impacts of SDoH in CVD, we can identify important intervention targets for populations most impacted by adverse social conditions on the path toward health equity in CVD outcomes. This is particularly pressing given the exacerbation of race/ethnic disparities in cardiometabolic disease and mortality resulting from the COVID-19 pandemic (3).

## Author contributions

All authors listed have made a substantial, direct, and intellectual contribution to the work and approved it for publication.

## Funding

The Powell-Wiley Lab was funded by the Division of Intramural Research of the National, Heart, Lung, and Blood Institute of the NIH and the Intramural Research Program of the National Institute on Minority Health and Health Disparities. The Maunakea lab was funded in part by the National Institute on Minority Health and Health Disparities (R01MD016593 and U54MD007601-34S2), the National Institute of Child Health and Human Development (OT2HD108105-01), and the National Institute of General Medical Sciences (P20GM139753). BN's work was funded by the National Institute on Minority Health and Health Disparities (R01MD011721 and R21MD012683).

## Conflict of interest

The authors declare that the research was conducted in the absence of any commercial or financial relationships that could be construed as a potential conflict of interest.

## Publisher's note

All claims expressed in this article are solely those of the authors and do not necessarily represent those of their affiliated organizations, or those of the publisher, the editors and the reviewers. Any product that may be evaluated in this article, or claim that may be made by its manufacturer, is not guaranteed or endorsed by the publisher.

## Author disclaimer

The views expressed in this manuscript are those of the authors and do not necessarily represent the views of the National Heart, Lung, and Blood Institute; the National Institute on Minority Health and Health Disparities; the National Institute of Child Health and Human Development; the National Institute of General Medical Sciences the National Institutes of Health; or the U.S. Department of Health and Human Services.
